# Combined Percutaneous and Transurethral Lithotripsy for Forgotten Ureteral Stents With Giant Encrustation

**DOI:** 10.5812/numonthly.9533

**Published:** 2013-06-01

**Authors:** Ibrahim Uygun

**Affiliations:** 1 Department of Pediatric Surgery and Pediatric Urology, Dicle University Medical Faculty, Diyarbakir, Turkey

**Keywords:** Kidney Calculi, Ureteroscopy, Laser, Lithotripsy, Stents

*Dear Editor,*

Forgotten ureteral stents (FUS) with encrustation can cause problems during treatment. Most FUS encrustations are small and are treated easily. However, if large, multiple, or long stones have formed, treatment is challenging to the surgeon. We congratulate Rabani, who reported successful treatment of a giant (> 35 mm stone formation) encrustation (1). As it is clearly known, patients with FUS refer to a doctor occasionally, but usually due to urosepsis or presepsis. Thus, their diagnosis should be sepsis, and their treatment should be carefully planned. Nevertheless, further evaluation and medical treatment should be conducted before the initial invasive procedure (2). After a physical examination, blood and urine analyses, plain X-rays, and ultrasonography should be performed. If the patient is in renal failure, it should be treated with urinary drainage by nephrostomy of the affected kidney and/or drainage of the contralateral kidney. A kidney evaluation (DMSA, DTPA, IVP, CT) and appropriate treatment should be performed. The management of encrusted FUS was reported in detail by Bostanci et al. (3).

We agree with Rabani that the best treatment is to prevent this complication and design a recall system (1). Thus, Sancaktutar et al. at our university reported a reminder short message service (SMS) based on a computer system that tracks ureteral stents and automatically sends a reminder to the mobile phones of patients and urologists using an integrated stent register program and a stent extraction reminder program with an electronic patient record program located within our hospital’s computer network. This system has been used successfully on several patients (4).

Treatment of encrusted FUS may require several combined endourological procedures (cystoscopic removal, ESWL, PCNL, URS, percutaneous or transurethral cystolithotripsy, open surgery, etc.). Rabani used percutaneous and transurethral cystolithotripsy with a bladder coil for a patient with a giant (> 35 mm) encrusted FUS and PCNL with a kidney coil using a pneumatic lithotripsy energy source. We recommend holmium laser lithotripsy in addition to pneumatic lithotripsy, which is effective and safe for treating urinary stones at all locations (5). However, laser energy can also break the ureteral stent.
